# Association between Chewing Problems and Sleep among Japanese Adults

**DOI:** 10.1155/2019/8196410

**Published:** 2019-12-17

**Authors:** Tetsuji Azuma, Koichiro Irie, Kazutoshi Watanabe, Fumiko Deguchi, Takao Kojima, Akihiro Obora, Takaaki Tomofuji

**Affiliations:** ^1^Department of Community Oral Health, School of Dentistry, Asahi University, 1851 Hozumi, Mizuho, Gifu 501-0296, Japan; ^2^Department of Microbiology and Immunology, Columbia University Medical Center, 701 West 168th Street, New York 10032, NY, USA; ^3^Department o f Oral Health and Preventive Dentistry, Meikai University School of Dentistry, Sakado, Saitama 350-0283, Japan; ^4^Asahi University Hospital, 3- 23 Hashimoto-cho, Mizuho, Gifu 500-8523, Japan

## Abstract

An association between physical illness and sleep has been suggested. Disordered chewing might be a physical factor that is associated with sleep issues. This cross-sectional study aimed to determine whether chewing problems are associated with sleep in Japanese adults. Sleep and chewing issues were evaluated in 6,025 community residents using a self-reported questionnaire. The prevalence of poor sleep quality and sleeping for <6 h/day (short duration) were 15.6% and 29.4%, respectively. Multivariate logistic regression analyses showed that prevalence of poor sleep quality was significantly associated with self-reported medical history (odds ratio (OR), 1.30; *p* < 0.001), self-reported symptoms (OR, 4.59; *p* < 0.001), chewing problems (OR, 1.65; *p* < 0.001), and poor glycemic control (OR, 1.43; *p*=0.035). The prevalence of short sleep duration was also significantly associated with female sex (OR, 1.23; *p*=0.001), self-reported symptoms (OR, 1.60; *p* < 0.001), chewing problems (OR, 1.30; *p*=0.001), and being overweight (OR, 1.41; *p* < 0.001). In conclusion, chewing problems were associated with poor sleep quality and short sleep duration among Japanese adults.

## 1. Introduction

Chewing is an important oral function in terms of physical health [1]. Dental problems often cause difficulties with chewing food. Such chewing problem may induce various inconveniences for the health of the whole body. For instance, clinical studies have reported that chewing quality is associated with mental stress markers, such as salivary cortisol levels [2–4]. It is feasible that chewing problem may be detrimental to mental health.

Problems with sleep such as poor quality, as well as long and short durations, are a major public health concern. A systematic review and meta-analysis have shown that increased hemoglobin A1c (HbA1c) is associated with poor sleep quality and short and long sleep durations in patients with type 2 diabetes [5]. Another meta-analysis significantly associated insufficient sleep with diabetes mellitus, hypertension, cardiovascular diseases, coronary heart disease, and obesity [6]. These findings indicate that sleep problems are closely associated with metabolic disorders. Recent studies have also suggested that problematic sleep is a risk for dementia [7] and systemic inflammation [8]. These findings indicate that avoiding sleep problems is important for health maintenance.

Some studies are available regarding the relationship between oral health and sleep. A cohort study found that older adults with <10 teeth are at higher risk for short and long sleep durations, compared with individuals with ≥20 teeth [9]. The findings of a cross-sectional study have suggested a positive association between sleep problems and gingival inflammation [10]. However, the relationship between chewing and sleep remains unclear.

The purpose of this cross-sectional study was to investigate the relationship between chewing quality and sleep. In Japan, the Ministry of Health, Labor and Welfare has recommended specific health check that focuses on metabolic syndrome [11]. This health check has a questionnaire regarding chewing problem. Therefore, the research on Japanese adults was convenient to collect information on chewing problem. In the present survey, we hypothesized that the risk of poor sleep quality and short sleep duration are associated with chewing problems among Japanese adults. The specific aims of this study were to evaluate the relationship between chewing problem and poor sleep quality and the relationship between chewing problem and short sleep duration after adjusting variables related to sleep, including age, sex, self-medical history, self-reported current symptoms, use of hypnotic drugs, exercise habits, smoking habits, drinking habits, body mass index (BMI), and glycemic control.

## 2. Materials and Methods

### 2.1. Study Population

This cross-sectional study enrolled 6,228 Japanese adult community residents aged ≥20 years who participated in health checks at Asahi University Hospital (Gifu, Japan) between March and November 2018. Exclusion criteria comprised using hypnotic drugs (*n* = 171) and missing information (*n* = 32). Accordingly, we analyzed data from 6,025 community residents, all of whom provided written informed consent to participate in this study. The Ethics Committee at Asahi University approved the study protocol (No. 30018).

### 2.2. Evaluation of Sleep

A questionnaire was used to evaluate sleep quality and duration. Information about sleep quality was obtained from answers to the question “Do you usually feel refreshed after sleep?” with possible answers of “yes” and “no” [12]. A “no” response was categorized as poor sleep quality. Information on sleep duration was also obtained from answers to the question, “How many hours do you usually sleep per day?” [12]. Sleep duration was categorized as <6 (short duration) or ≥6 h/day [13, 14].

### 2.3. Evaluation of Chewing

Information about problems with chewing was obtained using the question “Which of the following statements most accurately describes your ability to eat or chew food?” Possible responses were “I can eat anything,” “Sometimes it is difficult to chew because of dental problems, such as dental caries and periodontal disease,” or “I can hardly chew.” Respondents who answered “Sometimes it is difficult to chew because of dental problems, such as dental caries and periodontal disease” were considered to have chewing problems.

This question is a part of a questionnaire that is included in nationwide health checks. Spearman correlations in our preliminary study of 430 volunteers identified positive associations between chewing problems and the numbers of decayed (*r* = 0.148, *p*=0.002) and missing (*r* = 0.150, *p*=0.002) teeth.

### 2.4. Different Variables Related to Sleep

We collected information about age, sex, self-reported medical history (yes/no), self-reported current symptoms (yes/no), hypnotic drugs (yes/no), regular exercise (yes/no), smoking habit (yes/no), and alcohol consumption (yes/no).

The weight and height of all participants were measured using a body composition meter (Tanita, Tokyo, Japan), and BMI was calculated. A BMI ≥25 was defined as overweight [15]. Fasting serum HbA1c concentrations were determined using a DM-JACK diabetes automatic analyzer (Kyowa Medex, Tokyo, Japan). An HbA1c value of ≥6.5% was defined as poor glycemic control [16].

### 2.5. Statistical Analysis

Continuous variables are expressed as medians (first and third quartiles). Significant differences in selected characteristics between study participants with good and poor sleep quality and sleep duration were assessed using the chi-squared and Mann–Whitney *U* tests.

In this study, the primary outcome variables were sleep quality and duration. The predictor variable was the presence or absence of chewing problem. Univariate and stepwise multivariate logistic regression analyses proceeded using poor sleep quality and short sleep duration as dependent variables. As the third category of variables concerned the sample (age, sex, BMI, and glycemic control) and different variables related to sleep (self-medical history, self-reported current symptoms, use of hypnotic drugs, exercise habits, smoking habits, and drinking habits), these variables were adjusted in these analyses. Variables with *p* < 0.10 and *p* < 0.05 were, respectively, removed and added to the model. Independent variables with *p* < 0.05 in the univariate model were selected. Multicollinearity was avoided using Spearman correlation analyses between variables. Based on a previous study [17], variables with |*r*| > 0.8 in the Spearman correlation analysis were removed. However, the maximum value of the correlation coefficient in this study was *r* = 0.278. All data were analyzed using SPSS statistics version 25 (IBM Japan, Tokyo, Japan). Values with *p* < 0.05 were considered statistically significant.

## 3. Results

### 3.1. Prevalence of Participants with Different Sleep Quality, Sleep Duration, and Chewing Quality

Among the participants, 5,085 (84.4%) and 940 (15.6%) had good and poor sleep quality, respectively. The numbers of participants with sleep duration <6 and ≥6 h/day were 1,770 (29.4%) and 4,255 (70.6%), respectively. Furthermore, 5,198 (86.3%), 804 (13.3%), and 23 (0.4%) participants, respectively, answered “I can eat anything,” “Sometimes it is difficult to chew because of dental problems, such as dental caries and periodontal disease,” and “I can hardly chew.”

### 3.2. Characteristics of Participants with Different Sleep Quality and Duration

Respondents with poor sleep quality were significantly more likely to be older (*p*=0.007), have higher prevalence of self-reported medical history (*p* < 0.001), current symptoms (*p* < 0.001), chewing problems (*p* < 0.001), being overweight (*p*=0.045), poor glycemic control (*p*=0.007), and have a lower prevalence of regular exercise (*p*=0.03). Respondents with short sleep duration were also significantly more likely to be female (*p* < 0.001), have a higher prevalence of self-reported symptoms (*p* < 0.001), chewing problems (*p* < 0.001), being overweight (*p* < 0.001), have a lower prevalence of regular exercise (*p*=0.005), and alcohol consumption (*p*=0.011; Table 1).

### 3.3. Association between Chewing Problem and Poor Sleep Quality

Univariate logistic regression associated the odds ratio of poor sleep quality with age (OR, 1.01; *p*=0.012), self-reported medical history (OR, 1.47; *p* < 0.001), self-reported current symptoms (OR, 4.87; *p* < 0.001), chewing problem (OR, 1.84; *p* < 0.001), regular exercise (OR, 0.82; *p*=0.029), overweight (OR, 1.18; *p*=0.042), and poor glycemic control (OR, 1.58; *p*=0.005; Table 2). Multivariate logistic regression associated the odds ratio of poor sleep quality with self-reported medical history (OR, 1.30; *p* < 0.001), self-reported symptoms (OR, 4.59; *p* < 0.001), chewing problems (OR, 1.65; *p* < 0.001), and poor glycemic control (OR, 1.43; *p*=0.035) after adjusting for age, self-reported medical history, self-reported symptoms, chewing problems, regular exercise, being overweight, and poor glycemic control (Table 3).

### 3.4. Association between Chewing Problem and Short Sleep Duration

Univariate logistic regression analyses associated the odds ratio of short sleep duration with female sex (OR, 1.22; *p* < 0.001), self-reported current symptoms (OR, 1.69; *p* < 0.001), chewing problems (OR, 1.34; *p* < 0.001), regular exercise (OR, 0.81; *p*=0.005), alcohol consumption (OR, 0.82; *p*=0.011), and being overweight (OR, 1.34; *p* < 0.001; Table 4). Multivariate logistic regression analyses associated the odds ratio of short sleep duration with female sex (OR, 1.23; *p*=0.001), self-reported symptoms (OR, 1.60; *p* < 0.001), chewing problems (OR, 1.30; *p*=0.001), and being overweight (OR, 1.41; *p* < 0.001) after adjusting for sex, self-reported symptoms, chewing problems, regular exercise, alcohol consumption, and being overweight (Table 5).

## 4. Discussion

To the best of our knowledge, this is the first investigation of an association between sleep and chewing problems among Japanese adults. We found that chewing problems were significantly associated with poor sleep quality and short sleep duration after adjusting for the potentially confounding variables of self-reported current symptoms and exercise habits. These findings indicated that chewing problems could be a risk factor for poor sleep quality and short sleep duration. Therefore, improving chewing problems by improving dental health might contribute to better sleep.

Some studies have examined the relationship between oral health and sleep. A cross-sectional study of schoolchildren in Brazil has significantly associated probable sleep bruxism with poor sleep quality in children aged 8–10 years [18]. A cross-sectional study of a French population [11] and a case-control study of a Malaysian population [19] have also suggested that sleep issues are significantly associated with poor periodontal health. In addition, a Japanese cohort study has suggested that the risk for short and long sleep durations differs according to the numbers of present teeth [10]. Our findings of a significant association between sleep problems and poor oral health are consistent with these results.

The underlying mechanisms of the relationship between chewing problems and sleep remain unknown. However, some explanations are plausible. Chewing problems can interfere with healthy eating habits. Since inadequate eating habits increase risk for sleep problems [20], changes in eating habits due to chewing problems might be detrimental to achieving adequate sleep. Additionally, chewing is an effective stress-coping behavior [21]. Therefore, chewing problems reflecting chronic stress might be associated with sleep problems.

The present study associated poor glycemic control with poor sleep quality. Being overweight was also associated with short sleep duration. These observations are in agreement with those of previous studies showing that sleep problems are associated with diabetes mellitus [22] and a higher BMI [23]. In addition, female sex, symptoms of illness, and extant medical conditions are potential risk factors for sleep problems [13]. The present findings also associated risk of poor sleep quality and/or short sleep duration with being female, self-reported symptoms of illness, and self-reported medical history.

On the other hand, we found that poor sleep quality and short sleep duration were not associated with smoking habits, exercise habits, and alcohol consumption [24–26]. The prevalence of participants in the present study who were current smokers, participated regularly in exercise, and consumed alcohol daily was 20.2%, 13.3%, and 16.6%, respectively. However, these numbers were insufficient to conclude significant associations with sleep problems.

The prevalence of poor sleep quality in Western countries is 20–30%, and it increases with age [27]. In addition, 15–20% of adults have reported having chronic sleep problems [28]. Our findings were similar to these, as the overall prevalence of poor sleep quality and short sleep duration was 15.6% and 29.4%, respectively. In contrast, the prevalence of individuals who sleep >8 h/day was <0.1% in the present study. Therefore, we could not investigate the relationship between chewing problems and long sleep duration, which might limit the ability to extrapolate our findings to the general population.

Some potential limitations should be addressed. All participants were recruited from a single hospital, which might have resulted in overestimation or underestimation due to sampling bias. Variables including sleep quality, sleep duration, and chewing problems were self-reported. Finally, the cross-sectional study design did not allow us to conclude whether chewing problems are causes or effects of sleep problems. Longitudinal studies are necessary to clarify causality between chewing problems and sleep.

## 5. Conclusion

Chewing problems are associated with poor sleep quality and short sleep duration among Japanese adults. Chewing problems might be risk factors for sleep disorders.

## Figures and Tables

**Table 1:** Characteristics of participants according to sleep quality and duration.

Variables	Sleep quality			Sleep duration		
	Good (*n* = 5085)	Poor (*n* = 940)	*p* value	<6 h/day (*n* = 1770)	≥6 h/day (*n* = 4255)	*p* value
Sex^a^	2190 (43.1)	375 (39.9)	0.073	815 (46.0)	1750 (41.1)	<0.001
Age^b^	51 (45, 57)	52 (46, 57)	0.007	51 (46, 56)	51 (45, 57)	0.215
Self-reported medical history^c^	2378 (46.8)	529 (56.3)	<0.001	880 (49.7)	2027 (47.6)	0.149
Self-reported symptoms^c^	3750 (73.7)	876 (93.2)	<0.001	1468 (82.9)	3158 (74.7)	<0.001
Chewing problems^c^	632 (12.4)	195 (20.7)	<0.001	288 (16.3)	539 (12.7)	<0.001
Regular exercise^c^	1051 (20.7)	165 (17.6)	0.030	317 (17.9)	899 (21.1)	0.005
Smoking habit^c^	677 (13.3)	122 (13.0)	0.834	233 (13.2)	566 (13.3)	0.901
Alcohol consumption^c^	840 (16.5)	159 (16.9)	0.775	260 (14.7)	739 (17.4)	0.011
Overweight^c^	1175 (23.1)	246 (26.2)	0.045	486 (27.5)	935 (22.0)	<0.001
Poor glycemic control^c^	178 (3.5)	51 (5.4)	0.007	73 (4.1)	156 (3.7)	0.416

^a^Number of females (%); ^b^years, median (first and third quartiles); ^c^number of incidences (%). *p* values were calculated using the chi-squared and Mann–Whitney *U* tests.

**Table 2:** Univariate logistic regression analysis of factors associated with poor sleep quality.

Variables	Crude odds ratio	95% confidence interval	*p* value
Sex^a^	0.88	0.76–1.01	0.071
Age	1.01	1.00–1.02	0.012
Self-reported medical history^b^	1.47	1.27–1.69	<0.001
Self-reported symptoms^b^	4.87	3.75–6.33	<0.001
Chewing problems^b^	1.84	1.54–2.20	<0.001
Regular exercise^b^	0.82	0.68–0.98	0.029
Smoking habit^b^	0.97	0.79–1.19	0.718
Alcohol consumption^b^	1.03	0.85–1.24	0.768
Overweight^b^	1.18	1.01–1.38	0.042
Poor glycemic control^b^	1.58	1.15–2.18	0.005

^a^Female/male (reference was male); ^b^presence/absence (reference was absence).

**Table 3:** Stepwise multivariate logistic regression analysis of factors associated with poor sleep quality.

Variables	Adjusted odds ratio^a^	95% confidence interval	*p* value
Self-reported medical history^b^	1.30	1.13–1.51	<0.001
Self-reported symptoms^b^	4.59	3.53–5.97	<0.001
Chewing problems^b^	1.65	1.38–1.98	<0.001
Poor glycemic control^b^	1.43	1.03–1.99	0.035

^a^Adjusted for age, self-reported medical history, self-reported current symptoms, chewing problems, regular exercise, being overweight, and poor glycemic control; ^b^presence/absence (reference was absence).

**Table 4:** Univariate logistic regression analysis of factors associated with short sleep duration (<6 h/day).

Variables	Crude odds ratio	95% confidence interval	*p* value
Sex^a^	1.22	1.09–1.37	<0.001
Age	0.995	0.99–1.00	0.175
Self-reported medical history^b^	1.09	0.97–1.21	0.141
Self-reported symptoms^b^	1.69	1.47–1.95	<0.001
Chewing problems^b^	1.34	1.15–1.57	<0.001
Regular exercise^b^	0.81	0.70–0.94	0.005
Smoking habit^b^	0.99	0.84–1.16	0.885
Alcohol consumption^b^	0.82	0.70–0.96	0.011
Overweight^b^	1.34	1.18–1.53	<0.001
Poor glycemic control^b^	1.13	0.85–1.50	0.397

^a^Female/male (reference was male); ^b^presence/absence (reference was absence).

**Table 5:** Stepwise multivariate logistic regression analysis of factors associated with short sleep duration (<6 h/day).

Variables	Adjusted odds ratio^a^	95% confidence interval	*p* value
Sex^b^	1.23	1.09–1.38	0.001
Self-reported symptoms^c^	1.60	1.39–1.85	<0.001
Chewing problems^c^	1.30	1.11–1.52	0.001
Regular exercise^c^	0.88	0.76–1.01	0.075
Overweight^c^	1.41	1.23–1.60	<0.001

^a^Adjusted for sex, self-reported symptoms, chewing problems, regular exercise, alcohol consumption, and being overweight; ^b^female/male (reference was male); ^c^presence/absence (reference was absence).

## Data Availability

The data used to support the findings of this study are available from the corresponding author upon request.
